# Malignant Peripheral Nerve Sheath Tumor of the Cerebellar Hemisphere: An Unusual Location and Multiple Intracranial Parenchyma Metastases

**DOI:** 10.7759/cureus.14373

**Published:** 2021-04-08

**Authors:** Jiangwei Ding, Lei Wang, Haibiao Zhao, Feng Wang, Tao Sun

**Affiliations:** 1 Department of Neurosurgery, General Hospital of Ningxia Medical University, Ningxia Medical University, Yinchuan, CHN; 2 Department of Neurosurgery, Ningxia Key Laboratory of Cerebrocranial Disease, Ningxia Medical University, General Hospital of Ningxia Medical University, Yinchuan, CHN; 3 Department of Neurosurgery, The First Affiliated Hospital of Zhengzhou University, Zhengzhou, CHN; 4 Department of Neurosurgery, The First Affiliated Hospital, Zhejiang University School of Medicine, Hangzhou, CHN; 5 Department of Neurosurgery, Ningxia Key Laboratory of Cerebrocranial Disease, General Hospital of Ningxia Medical University, Yinchuan, CHN

**Keywords:** malignant peripheral nerve sheath tumors, malignant intracerebral nerve sheath tumors, brain parenchyma metastases, cerebellar mpnst

## Abstract

Malignant peripheral nerve sheath tumors (MPNSTs) are rare soft tissue malignancies that can occur in any part of the body. The most common sites are the proximal limbs and trunk. Intracranial MPNSTs are rare; most originate from the auditory, trigeminal, and other cranial nerves, and occurrence within the brain parenchyma is rarer. Here, we describe a malignant peripheral schwannoma in the cerebellar hemisphere of the brain parenchyma. To our knowledge, this is the first case of brain parenchymal metastasis of an MPNST. We observed no effects on the tumor after the application of multiple chemotherapy drugs; thereafter, we explored the literature surrounding the condition.

## Introduction

Malignant peripheral nerve sheath tumors (MPNSTs) are rare soft tissue sarcomas, often associated with neurofibromatosis type-1 [1-2]. In addition, the incidence of MPNST is high in patients with a history of radiation therapy [3]. An intracranial MPNST is rare even in patients with multiple intracranial metastases, and the prognosis is very poor (associated with high mortality).

## Case presentation

**Figure 1:**
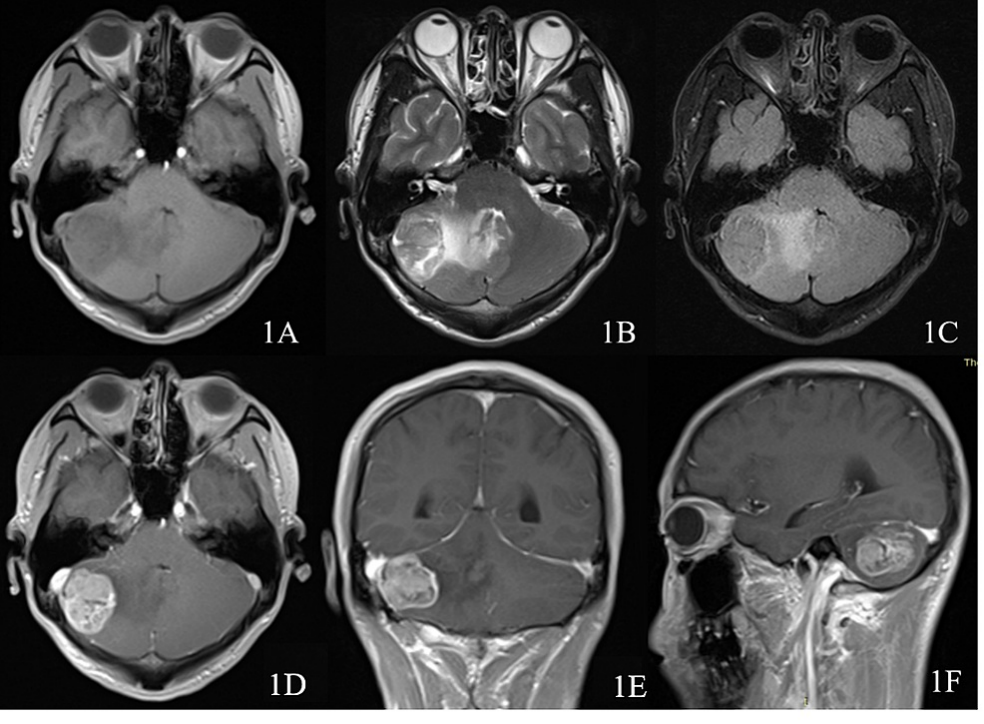
Preoperative examination Magnetic resonance imaging (MRI) scan showing tumor that is hypointense on T1WI, mixed hyperintense on T2WI, and iso-signal on fluid-attenuated inversion-recovery (1A-1C). Postgadolinium scan showing a significant but heterogeneous enhancement mass 35.4 mm × 30.5 mm × 30.3 mm (1D-1F).

A 37-year-old female with no signs of von Recklinghausen disease presented with a history of headache and dizziness of 20 days duration. Neurological examination showed ataxia (the left side of both hands alternating movement test, heel-knee-tibia test, and finger-nose test could not be completed). No notable abnormalities were observed on other nervous system examinations. A magnetic resonance imaging (MRI) brain scan showed a lesion in the right cerebellar hemisphere. The tumor was long T1 and mixed long T2 signal intense, with an unclear boundary and equal signal of diffusion-weighted imaging high b value. Post-gadolinium scan showed a significant but heterogeneous enhancement mass with a dimension of 35.4 mm × 30.5 mm × 30.0 mm and clear boundary. The preoperative diagnosis was meningioma (Figure 1).

**Figure 2:**
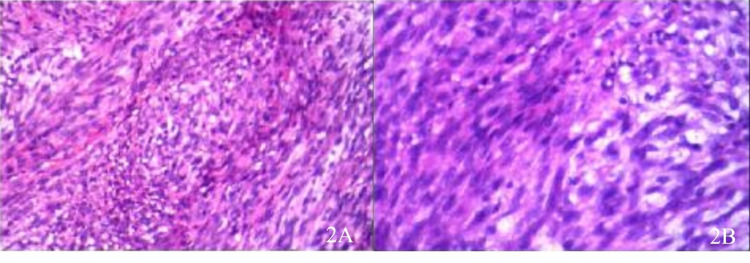
Postoperative histopathology: the cells are arranged in bundles and whirlpools, the majority of the tumors are spindle-shaped eosinophilic, and the cell boundary is unclear (HE × 200) HE: hematoxylin & eosin

A right midline incision with a right-sided paramedian approach craniotomy was performed. Under the microscope, the tumor tissue was gray-yellow without a capsule and had a clear boundary from the surrounding brain tissues. The adhesion between the tumor surface and the tentorial surface was significant. Intraoperative differential diagnosis was a likely metastatic tumor rather than meningioma. The tumor was completely resected. Pathological examination showed a large number of spindle cells, arranged in swirls and bundles, with abundant eosinophilia (Figure 2).

Immunohistochemistry showed that tumor cells were positive for CKA1, vimentin, and S-100 but lacked reactivity to nestin, epithelial membrane antigen, smooth muscle actin, and glial fibrillary acid protein. The Ki-67 labeling index was 70%. This is consistent with a malignant peripheral nerve sheath tumor.

**Figure 3:**
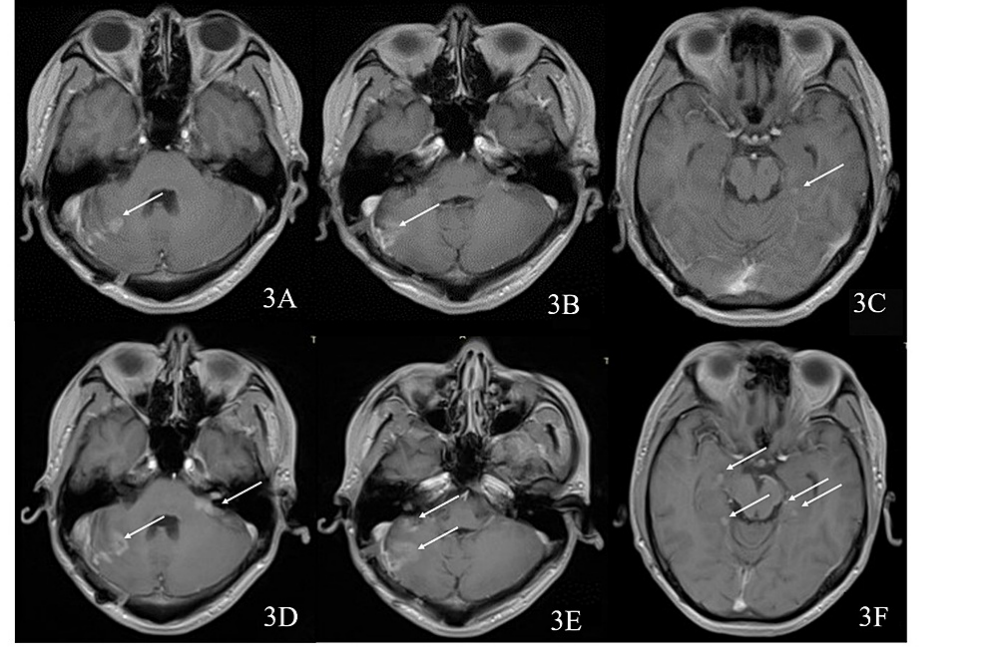
Postoperative MRI scans At 15 months after the operation, a magnetic resonance imaging (MRI) scan showing patchy enhancement in the right cerebellar hemisphere, which is adjacent to meningeal enhancement (3A-3C), and punctate enhancement signal in the left hippocampus. MRI at 17 months postoperative showing the lesions in the right cerebellar hemisphere to be more extensive than before, and the newly developed left brachium pontis, left cerebellopontine angle area, left auditory nerve trunk, and bilateral hippocampus areas to be abnormally enhanced (3D-3F).

After the operation, adjuvant whole-brain radiotherapy and cerebellar photon knife radiotherapy were performed, and six cycles of "formosetine" chemotherapy were carried out. Fifteen months postoperatively, the patient underwent surveillance MRI with a concern of recurrence, it was found that the lesions were more extensive than before, and tumor recurrence was considered (Figure 3, panels A-C).

**Figure 4:**
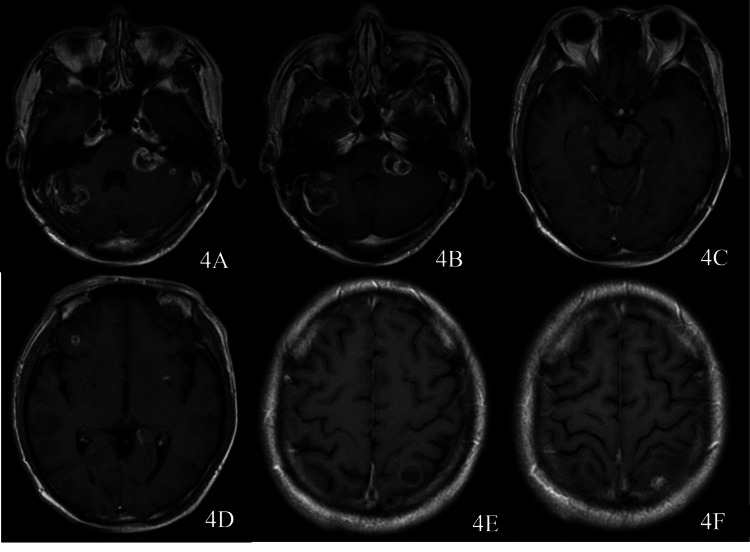
Findings at two years after the operation A magnetic resonance imaging (MRI) scan showing the tumors in the right cerebellar hemisphere (4A, 4B), the left brachium pontis, and the left cerebellopontine angle area to be substantially enlarged, and the ring to be enhanced (4A, 4B). The lesion of the left auditory nerve trunk is remarkably enlarged, the lesions in the right hippocampal area are slightly changed (4C), the enhanced signals in the left hippocampal area have disappeared, and new lesions can be seen in the right frontal lobe, left basal ganglia (4D), and left occipital lobe (4E, 4F).

In order to inhibit tumor growth, two cycles of "irinotecan" chemotherapy regimen were administered, and endu (recombinant human vascular endothelial inhibitor) was administered to inhibit tumor vascular endothelial proliferation. At the same time, "methotrexate 15 mg + dexamethasone 5 mg" was injected intrathecally. Seventeen months post the operation, MRI showed that the lesion was more extensive than before (Figure 3, panels D-F). Two cycles of chemotherapy were carried out with the regimen of "formosetine + vincristine + prednisone." MRI results showed the recurrence of the lesion two years after the operation (Figure 4).

## Discussion

MPNSTs are malignant neoplasms accounting for only 3-5% of soft tissue sarcomas [1]. Neurofibromatosis type-1 (NF1) patients have a cumulative lifetime risk of developing MPNST of 8-16%, and the predilection age is 20-35 years [2]. MPNST is more common in the trunk and extremities but rare in the brain, where it is also called malignant intracerebral nerve sheath tumor (MINST) [4]. Enrico Martin et al. analyzed 3267 cases of MPNSTs of which 2329 cases (71.3%) were in the trunk and limbs, and 167 cases (5.1%) were in the cranium [5]. MPNSTs are extremely rare in the cerebellum; only two cases (including this case) of cerebellar hemisphere MPNST and one case of cerebellar vermis MPNST have been reported in the literature [6-7] (Table 1).

**Table 1:** Literature review Abbreviations: F, female; NA, not available; GTR, gross total resection; PR, partial resection; VP shunt, ventriculoperitoneal shunt; m, months; y, years; RT, right; RAD, radiotherapy; CHE, chemotherapy

Case	Study	Sex	Age（y）	Location	Presenting Complain	NF-1	Treatment	Recurrence	Metastases	Ki-67	Follow-up
1	Singh et al [6] (1993)	F	61	RT cerebellar hemisphere	Headache, vomiting, increasing unsteadiness of gait, and diplopia	NO	GTR, RAD, VP shunt (ventricular dilatation 10 months after operation)	8m	NO	NR	18m died
2	Maiuri et al [7](2004)	F	29	Cerebellar vermis	Headache, vomiting, dizziness, transient loss of vision, and difﬁculty in walking.	NO	PR + RAD（50Gy ）	6m	NO	NR	8m died
3	Present case	F	37	RT cerebellar hemisphere	Headache and dizziness,	NO	GTR, RAD, CHE	15m	Multiple intracranial Parenchyma	70%	24m alive

Intracranial MPNSTs are mainly located in the vestibular nerve (about 50-60% of all cranial nerve MPNST), followed by the vagus, trigeminal, and facial nerves [8]. The clinical manifestations of intracranial MPNST are related to its location and size. Shutran et al. reported a case of trigeminal MPNST with corpus callosum metastasis with trigeminal neuralgia as the first symptom [9]. The main manifestation of MPNST in brain parenchyma is intracranial hypertension [10].

MRI is the first choice of investigation for the diagnosis of MPNST. The main manifestations of MRI are mixed-signal intensity, irregular contour with necrosis, compression of adjacent brain tissue by the lesions, significant local invasion, and signal enhancement in almost all cases. A computed tomography scan may show tumor invasion of surrounding bone [11]. Chen L et al. reported two MRI scans of MPNST in the cerebellopontine angle area: low signal on T1WI, mixed high signal on T2WI, and significant inhomogeneous enhancement [12]. The clinical manifestations of this case were headache, dizziness, and ataxia. The imaging features were long T1 mixed with long T2 signals, and the lesion was inhomogeneous and significantly enhanced, which is consistent with the findings of the literature.

The etiology of MINST is still controversial. At present, it is believed that there is a correlation between the tumor and neurofibromatosis and radiation. Chica et al. reported a 38-year-old MINST patient who received radiotherapy for Hodgkin's lymphoma during childhood [4]. Baehring et al. found MPNST in three out of 54 patients, and these three patients had a history of radiotherapy [13]. There are several points about the origin of MINST unrelated to the cranial nerve: (1) since there are no Schwann cells in the central nervous system, some scientists believe that the tumor originates from the Schwann cells of the peripheral nerves of blood vessels or other stromal cells; (2) adrenergic nerve fibers distributed in the arterioles are also thought to be the origin; (3) meningeal branches of the trigeminal nerve have been postulated to be the origin in some cases; and (4) neural crest cells in the embryo early fetal migration may also be one of the sources of the origin of the tumor [13-14].

The most common location of metastasis is the lung [15] while brain metastasis is extremely rare. In 2016, Puffer et al. reported only 21 cases of brain metastasis [16]. Most of the reported cases were extracranial MPNST metastasis to the brain, but there are few reports on the intracerebral metastasis of MINST. L'heureux-Lebeau et al. analyzed 60 MINST cases and reported that 19% of them were accompanied by metastasis, among which three cases were considered to have cerebrospinal fluid dissemination and metastasis [17]. Shutran et al. reported a case of MINST, which originated from the malignant peripheral neurinoma of the trigeminal nerve and transferred to the corpus callosum. MPNST of the spine may have intracranial and leptomeningeal metastasis [9]. Baharvahdat et al. reported 23 cases of primary spinal malignant peripheral neurilemmoma and found six cases with brain metastasis [18]. To our knowledge, this report is the first case of a cerebellar hemisphere MPNST with multiple intracerebral metastases. It has been reported that the expression of Ki-67 in tumor cells is associated with a poor prognosis [19]. L'heureux-Lebeau et al. reported that the one-year survival rate of MINST was only 33%, 45% of cases showed recurrence, and 33% had metastasis [17].

Complete surgical resection is the standard treatment for MPNST [20]. Postoperative radiotherapy is the main adjuvant treatment. It is reported that postoperative radiotherapy can improve survival rate and prognosis. The mortality rate with radiotherapy was 65% while, without radiotherapy, it was 79%, with a one-year survival rate of 65% for the seven survivors who received radiotherapy [17]. It is reported that combination chemotherapy is effective for the treatment of MPNST. Generally, the combination of ifosfamide, etoposide, and anthracycline as chemotherapy drugs is used, but the overall remission rate is relatively low [10]. The selection and effect of chemotherapy drugs still need further study. Since the tumor is rare, there is no standard therapeutic schedule.

Intracranial MPNST is relatively rare in clinical practice. It is difficult to differentiate them from benign sheath tumors on imaging, as there are no significant image features. It is often diagnosed as meningioma or benign schwannoma before the operation, and it needs to be confirmed by pathology and immunohistochemistry. MPNST is highly malignant with a poor prognosis.

## Conclusions

MPNST often occurs in other body parts, but it is rare intracranially. Despite the combined treatment of surgery, radiotherapy, and chemotherapy, the prognosis is still very poor. We report a case of cerebellar MPNST unrelated to cranial nerves with multiple brain parenchyma metastases; this is the first of such reports to our knowledge.
